# Retrospective Audit of Transfusion Reactions in a Tertiary-Care Hospital in South India

**DOI:** 10.7759/cureus.74930

**Published:** 2024-12-01

**Authors:** Sabari Priya, Karthikeyan V, Sowmya Srinivasan

**Affiliations:** 1 Transfusion Medicine, Mahatma Gandhi Medical College and Research Institute, Pondicherry, IND; 2 Pathology, Mahatma Gandhi Medical College and Research Institute, Pondicherry, IND

**Keywords:** blood component therapy, haemovigilance, hemovigilance, monitoring, patient care, patient safety, reporting, transfusion reactions

## Abstract

Background: Blood transfusion is a double-edged sword, as it is a life-saving intervention but is also associated with various adverse reactions. However, blood transfusion safety can be improved by identifying these adverse reactions and taking appropriate interventions. Therefore, in this study, we aimed to determine the frequency and type of transfusion reactions occurring among in-patients at our hospital, as reported to our institute's blood center.

Materials and methods: This cross-sectional study was carried out at the blood center of a tertiary-care hospital over three years and nine months from 2021 to 2024. During the study period, all transfusion reactions related to various blood and blood products reported to our blood center were recorded and analyzed according to departmental standard operating procedures.

Results: Out of 23,028 units of blood and blood products transfused during the study period, 105 (0.5%) cases of transfusion reaction were documented. The most common transfusion reaction reported was febrile non-hemolytic transfusion reaction (n=62; 59%), followed by allergic transfusion reaction (n=33; 31%), and 10 (10%) reported reactions were due to various other reasons.

Conclusion: The relatively low incidence of transfusion reactions in this study (0.5%) may be due to our study's underreporting of transfusion reactions. This difference highlights the need to conduct continuous medical, educational programs for all healthcare professionals involved in the transfusion chain. Such programs should highlight the importance of documentation and reporting of adverse transfusion reactions so that hemovigilance systems can be established for improved patient care.

## Introduction

Transfusion of blood and blood components is a life-saving procedure in clinical practice. Every year, millions of blood components are transfused globally. However, despite the benefits of transfusion of blood and blood products, adverse reactions also have been reported [1]. Adverse transfusion reaction, or simply transfusion reaction, is defined as an unwanted response or consequence in a patient temporarily correlated with administering blood and blood products [1]. The severity and nature of transfusion reactions depend on patient susceptibility and the specific blood products transfused [2,3].

According to the Hemovigilance Programme of India (HvPI), transfusion reactions can be broadly classified as acute (onset during or within 24 hours of transfusion) or delayed (onset after 24 hours up to 28 days of transfusion) depending on the time of occurrence, as well as immune or non-immune depending on pathophysiology. The literature estimates the incidence of transfusion reactions to be 0.001% to 10% [4]. However, these values are at risk of underestimation due to various reasons, including lack of knowledge and awareness of various transfusion reactions; ignoring minor allergic reactions and continuing transfusion after giving the patient anti-histamines; overlapping of signs and symptoms with the patient's underlying disease condition; difficulty in identifying reactions in unconscious patients; and lack of awareness to monitor for delayed transfusion reactions [5].

As a quality indicator of transfusion medicine, Hemovigilance functions as a continuous system of data collection for analysis of transfusion-related adverse events [6,7] and serves as a backbone of quality assurance. Adequate knowledge and awareness of the frequency and type of transfusion reactions aid in their timely identification, management, and treatment and prevent their occurrence or recurrence [6]. The present study was conducted to evaluate the pattern and frequency of adverse transfusion reactions in patients among various specialties in our hospital.

## Materials and methods

This was an ambidirectional study carried out over three years and nine months at the Department of Transfusion Medicine and Blood Center at a tertiary-care hospital in South India.

Ethical clearance to conduct the study was obtained from the Institutional Human Ethics Committee (Project No. MGMCRI/2024/01/04/IHEC/07). Consent for blood transfusion was obtained from patients or their attenders after explaining to them the benefits, risks, and alternatives of transfusion. Every patient was monitored during every transfusion from beginning to end and 24 hours post-transfusion. If transfusions were uneventful, transfusion audit forms were filed mentioning starting and ending times, and the empty blood bag was returned to the blood bank for autoclaving and discarding for traceability purposes.

The study duration was three years and nine months. During that time, data regarding transfusion reactions were obtained retrospectively from January 1, 2021, to February 29, 2024. After obtaining a consent waiver from the ethics review board, data was collected prospectively from March 1, 2024, to September 30, 2024. All transfusion reactions occurring among our hospital's patients reported to our blood center during the study period were included in the study. All cases admitted to our hospital with a history of outside transfusion reactions were excluded from the study.

Investigations of transfusion reactions

A protocol regarding how to proceed with clinical and laboratory investigations was framed according to guidelines laid down by the HvPI. Whenever a transfusion reaction was reported, the transfusion was stopped immediately, and the transfusion reaction was reported to the treating physician and a blood bank consultant.

After analyzing the details of the transfusion reactions and stabilizing the patient, the implicated blood, bag-along with the transfusion set, ethylenediamine tetraacetic acid, clotted sample, and details of the reaction documented in a transfusion reaction form-were submitted to the blood bank for further investigations. The details documented in the transfusion reaction notification form are described below.

Patient details

The transfusion reaction notification form included the following patient details: patient name, in-patient/out-patient number, age, sex, bed number/ward number, blood group, primary diagnosis, indication of transfusion, previous history of transfusion, and medication details.

Transfusion details

The transfusion reaction notification form included the following transfusion details: blood bag unit number, blood group, type of component, transfusion start and end times, rate of transfusion, the quantity of blood transfused, details of previous transfusions, details of anesthesia, and transfusion was given pre-op/intra-op/post-op or with non-surgical indication.

Transfusion reaction details

The transfusion reaction notification form included the following transfusion reaction details: pre-transfusion vitals and vitals at the time of reaction (temperature, pulse, blood pressure, respiratory rate, SpO2), relevant signs and symptoms (generalized, pain, renal, circulatory, respiratory), details of investigations undertaken bedside, investigations sent to other labs, clerical errors checked by comparing details of the implicated unit with that of patient details, management details, and reaction outcomes.

Blood bank investigations

As a part of blood bank investigations, patient details were checked against the blood bank crossmatch register, blood grouping register, issue register, and blood request register. Blood bags/transfusion sets were visually inspected for any abnormal mass or clot (if present). The patient's serum/plasma supernatant from pre-transfusion and post-transfusion and the returned blood bag sample were examined for hemolysis, discoloration, and clot. Pre-transfusion and post-transfusion samples and blood bags were analyzed for repeat blood grouping, crossmatching, and direct and indirect Coombs testing.

Evidence of hemolysis (in cases of suspected hemolytic transfusion reactions)

The following investigations were performed: plasma hemolysis (pre-transfusion and post-transfusion), urine hemoglobin, urine hemosiderin, serum bilirubin (pre-transfusion and post-transfusion), serum lactate dehydrogenase, coagulation workup (prothrombin time and activated partial thromboplastin time, full blood count, blood culture (blood bag and patient), and peripheral blood smear (Leishman stain and Gram stain). Based on the transfusion recipients' clinical features and laboratory parameters, transfusion reactions were classified according to the HvPI. The imputability levels of transfusion reactions were assessed by the blood center in coordination with attending physicians of the respective medical wards. Details of transfusion reactions were entered in Hemo-vigil software.

Statistical analysis

All parameters were entered into a spreadsheet with Microsoft Excel, and frequencies and percentages of categorial variables were analyzed.

## Results

During the study period, a total of 23,028 units of blood and blood components were transfused to our patients, comprising 11 (0.05%) units of whole blood, 9344 (40.58%) units of packed red cells, 8501 (36.92%) units of fresh frozen plasma, 543 (21.89%) units of platelets, and 129 (0.56%) units of cryoprecipitate (Table 1). There were a total of 105 (0.5%) documented transfusion reactions, with a higher incidence among males (n=53; 50.5%) than females (n=52; 49.5%) (Table 2).

**Table 1:** Distribution of total components issued

Serial number	Components	No of issues	Percentage
1	Whole blood	11	0.05%
2	Packed red cells	9344	40.58%
3	Fresh frozen plasma	8501	36.92%
4	Platelets	5043	21.89%
5	Cryoprecipitate	129	0.56%
Total		23,028	100%

**Table 2:** Gender-wise distribution of transfusion reactions

Year	Male	Female	n
2021	16	17	33 (31%)
2022	12	10	22 (21%)
2023	12	7	19 (18%)
2024	13	18	31 (30%)
Total n (%)	53 (50.5%)	52 (49.5%)	105

Incidence of transfusion reactions was higher among individuals 41-50 years of age (n=30; 29%) (Table 3). In addition, the incidence was higher among single-unit-transfused individuals (n=54; 51.4%) as compared to multi-unit-transfused individuals (n=51; 48.6%). The majority of transfusion reactions occurred in individuals with blood group B (n=40; 38%), followed by those with blood groups O (n=37; 35%) and A (n=21; 20%). Individuals with blood group AB had the lowest incidence of transfusion reaction (n=7; 7%) (Table 4).

**Table 3:** Age-wise distribution of transfusion reactions

Age (years)	n (%)
<20	9 (9%)
21–30	8 (8%)
31–40	15 (14%)
41–50	30 (29%)
51–60	16 (15%)
61–70	13 (12%)
71–80	13 (12%)
>80	1 (1%)

**Table 4:** Blood group-wise distribution of transfusion reactions by year

Blood group	2019	2020	2021	2022	n (%)
Group A	7	6	3	5	21 (20%)
Group B	16	8	8	8	40 (38%)
Group AB	2	1	1	3	7 (7%)
Group O	8	7	7	15	37 (35%)
Total	33 (31%)	22 (21%)	19 (18%)	31 (30%)	105

**Figure 1:**
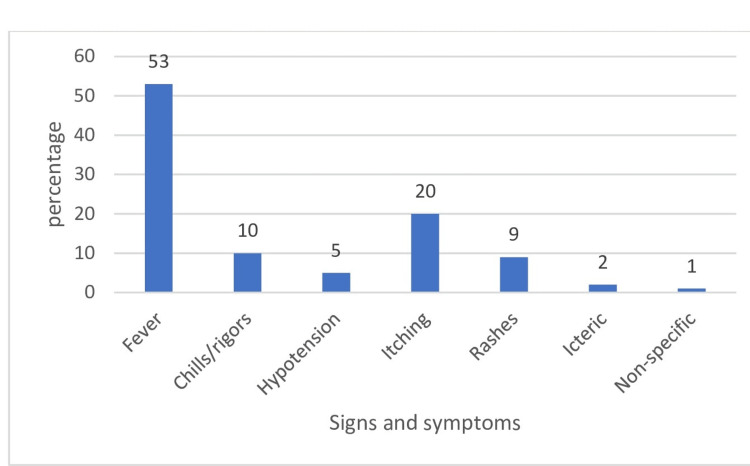
Distribution of signs and symptoms of transfusion reactions.

The most common type of transfusion reaction reported during the study period was febrile non-hemolytic transfusion reactions (n=62, 59%), followed by allergic transfusion reactions (n=33, 31%). Ten (10%) reported transfusion reactions were due to other reasons (Table 5). Frequencies of different signs and symptoms of transfusion reactions are depicted in Figure 1.

**Table 5:** Distribution of transfusion reaction types SI -serial number

SI	Reaction type	n (%)
1	Febrile transfusion reaction	62 (59%)
2	Allergic transfusion reaction	33 (31%)
3	Transfusion-associated circulatory overload	3 (3%)
4	Anaphylactic reaction	1 (1%)
5	Transfusion-associated hypotension	2 (2%)
6	Delayed transfusion reaction	2 (2%)
7	Non-specific	2 (2%)

Packed red blood cells were the most common component implicated in transfusion reactions, followed by platelets. The lowest incidence of transfusion reaction occurred with fresh frozen plasma and whole blood, and no reactions were reported with cryoprecipitate (Table 6).

**Table 6:** Relative frequency of transfusion reaction with different blood components. WB: whole blood, PRBCs: packed red blood cells, FFP: fresh frozen plasma, PC: platelet concentrate, Cryo: cryoprecipitate.

Reaction type	WB	PRBCs	FFP	PC	Cryo	Total
Febrile transfusion reaction	0	59	1	2	0	62
Allergic transfusion reaction	0	13	5	15	0	33
Transfusion-associated circulatory overload	0	3	0	0	0	3
Anaphylactic reaction	0	0	0	1	0	1
Transfusion-associated hypotension	0	2	0	0	0	2
Delayed transfusion reaction	0	2	0	0	0	2
Non-specific	1	0	0	1	0	2
Total	1	79	6	19	0	105

**Figure 2:**
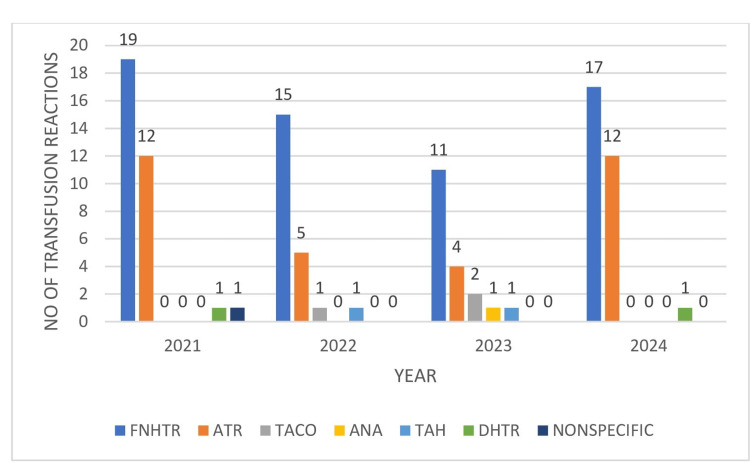
Transfusion reaction trends for the years 2021–2024 FNHTR: febrile non-hemolytic transfusion reaction, ATR: allergic transfusion reaction, ANA: anaphylactic reaction, TAH: transfusion-associated hypotension, DHTR: delayed hemolytic transfusion reaction, TACO: transfusion-associated circulatory overload.

Figure 2 presents the overall trends of transfusion reactions recorded during the study period (2021-2024), with the highest incidence of transfusion reactions occurring in 2021.

**Figure 3:**
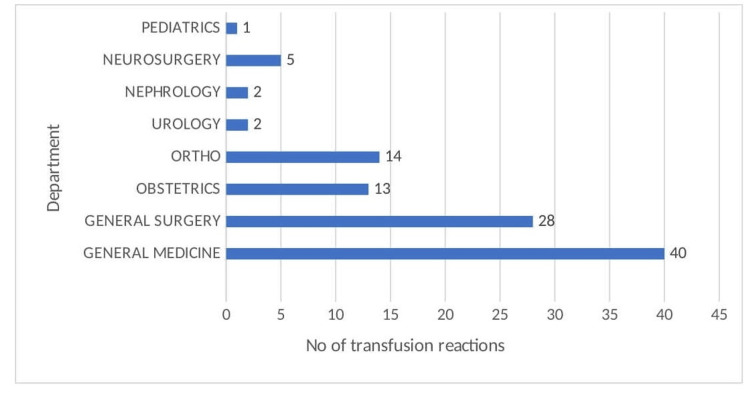
Department-wise distribution of transfusion reactions

In terms of specialty, the highest number of transfusion reactions was reported by the Department of Medicine, followed by the Department of General Surgery, as seen in Figure 3.

## Discussion

The reported incidence of transfusion reactions in the literature varies from 0.1% to 10.5% (Table 7). In the present study, the incidence of transfusion reaction was 0.5%, which was similar to the results reported by Kar et al. [1], Kumar et al. [7], Choudhury et al. [8], Gotekar et al. [9], Anitha et al. [10], Adjei et al. [11], Yangdon et al. [12], Krishnappa et al. [13], Chakravarty-Vartak et al. [14], and Bhattacharya et al. [15]; and much lower than that reported by Venkatachalapathy et al.(3.3%) [16], Pedrosa et al.(3.8%) [17], and Lubart et al.(10.5%) [18]. The low incidence we observed could be due to underreporting of transfusion reactions, lack of understanding/awareness of different transfusion reactions among new cadres of clinicians and staff, allergic transfusion reactions continued after giving anti-histamines, fear of punishment, and lack of experts/expertise on hemovigilance. These various causes of underreporting should be carefully analyzed in future studies.

**Table 7:** Incidence of transfusion reaction among various studies in the literature SI: Serial number

SI	Study authors	Incidence
1	Kar et al. (2021) [1]	0.09%
2	Kumar et al. (2013) [7]	0.05%
3	Chodhury et al. (2024) [8]	0.11%
4	Gotekar et al. (2020) [9]	0.2%
5	Anitha et al. (2023) [10]	0.48%
6	Adjei et al. (2024) [11]	0.51%
7	Yangdon et al. (2020) [12]	0.6%
8	Krishnappa et al. (2019) [13]	0.71%
9	Chakravarty et al. (2016) [14]	0.16%
10	Bhattacharya et al. (2011) [15]	0.18%
11	Present study	0.5%

In the current cross-sectional study, the incidence of transfusion reactions was higher among males (50.5%) compared to females (49.5%), similar to studies by Kar et al. [1] and Kumar et al. [7]. In contrast, in a study by Yangdon et al. [12], the incidence of transfusion reactions was higher among females compared to males. The higher incidence among males in our study may be due to the higher proportion of male transfusion recipients.

Multiple blood transfusions can increase the risk of transfusion reactions. However, in our study, the incidence of transfusion reactions was higher among recipients of single-unit transfusion (54.4%) than among those who received multiple transfusions. This result contrasts with studies by Ghataliya et al. [4], Venkatachlapathy et al. [16], and Bhattacharya et al. [15].

The current study reported the highest number of transfusion reactions among those with blood group B, followed by those with blood group O. This result contrasts with a study by Sinha et al. [19], where transfusion reactions were more common among those with blood group A, followed by those with blood group B. In a study by Anitha et al. [10], transfusion reactions were higher among those with blood group O, followed by those with blood group A.

In our study, transfusion reaction was more common among transfusion recipients 41-50 years of age. This finding is in contrast with studies by Choudhury et al. [8] and Somagari et al. [20], in which transfusion reaction was more common among transfusion recipients between 21 and 30 years of age. In our study, the incidence of transfusion reaction was higher in adults than in children, in contrast with a study by Allisabanair et al. [21] in which children showed a higher incidence of transfusion reaction.

Of all transfusion reactions reported, 76% occurred due to transfusion of packed red cells or whole blood. This rate to those reported by Sinha et al. [19] and Haslina et al. [22], is much higher than those reported by Kumar et al. and Payandeh et al. [23] and much lower than those reported by Adjei et al. [11], Venkatachalapathy [16], and Pahuja et al. [24] (Table 8).

**Table 8:** Comparison of transfusion reactions among various studies in relation to components transfused. PRBCs: Packed red cells

Study authors	Whole blood and PRBCs (%)	Fresh frozen plasma (%)	Platelets (%)
Kumar et al. (2013) [7]	42.8	37.75	19.38
Chodhury et al. (2024) [8]	100	0	0
Gotekar et al. (2020) [9]	77.12	1.2	16.88
Adjei et al. (2024) [11]	96.7	3.3	0
Bhattacharya et al. (2011) [15]	82.8	11.4	5.7
Venkatachalapathy et al. (2012) [16]	95.83	-	2.08
Sinha et al. (2016) [19]	68.3	12.98	18.18
Haslina et al. (2012) [22]	76.5	6.57	16.9
Payandeh et al. (2013) [23]	45.7	20.3	30.15
Pahuja et al. (2017) [24]	93.63	3.82	2.54
Present study	76	6	18

Platelets accounted for approximately 18% of transfusion reactions in the current study, similar to rates reported by Sinha et al. (18.18%) [19] and Kumar et al. (19.38%), and much lower than that reported by Payandeh et al. (30.15%) [23].

The most common transfusion reaction reported in our study was a febrile non-hemolytic transfusion reaction (n=62; 59%) caused by packed cell transfusion (n=59), platelets (n=2), and fresh frozen plasma (n=1). This rate is similar to those reported in studies by Yangdon et al. (59.2%) [12], Pai et al. (51.4%) [25], and Bassi et al. (50.9%) [26] and higher than those reported by Kumar et al. (35.7%) and Krishnappa et al. (31%) [13]. This high variation in the frequency of febrile non-hemolytic transfusion reactions can be attributed to differences in reporting systems, use of pre-medications (antipyretics), and the pre-transfusion condition of patients. Febrile transfusion reactions can be prevented by using leucocyte-reduced blood and blood products. As part of the transfusion reaction workup in the current study, remnants of blood bags sent for culture did not exhibit the growth of any microorganisms.

The second-most common reaction reported in the current study was allergic transfusion reaction (n=33; 31%) caused by platelets (n=15), packed red blood cells (n=13), and fresh frozen plasma (n=5). This incidence was much lower than those reported in studies by Sharma et al. (65.6%) [6], Kumar et al. (55.3%) [7], and Payandeh et al. (49.2%) [23]. The most common symptoms reported were urticaria, rash, and flushing, which subsided following anti-histamine administration and steroids. The lower incidence of allergic reactions in the current study may be due to underreporting of allergic reactions in the current study compared to other studies, as transfusion was continued after anti-histamine administration. In addition, strict donor screening strategies were followed, such as avoiding blood donations from donors with a known history of dust, pollen, or food allergy.

In the current study, only one case (1%) of the anaphylactic reaction occurred, representing a much lower incidence compared to those reported by Kumar et al. (5.1%) [7] and Gotekar et al. (2.59%) [9]. The reaction occurred following a platelet transfusion, during which the patient developed dyspnea and hypotension.

The current study reported transfusion-associated circulatory overload (TACO) in three (3%) cases following transfusion with packed red cells. In one case, the patient received a multiple-component transfusion; in another, the transfusion rate was high. Rapid transfusion of blood products must be avoided. A similar incidence of TACO was reported by Anitha et al. [10]. TACO following small-volume and single-unit transfusions have been reported in the literature [27].

In the current study, transfusion-associated hypotension, defined as a drop in blood pressure by more than 30 mmHg during or within one hour of a blood transfusion, was reported in two (2%) transfusion recipients; in both cases, the reaction occurred intraoperatively, and once transfusion was stopped, the patients recovered. This incidence was similar to those reported in studies by Bassi et al. (1%) [26] and Khalid et al. (1.4%) [28] and much lower than that reported by Payandeh et al. (6.8%) [23]. 

Delayed hemolytic transfusion reaction was reported in two(2%) patients, both of whom received multiple-component transfusion; one of the patients had post-transfusion raised bilirubin, and in another patient, icterus was noticed post-transfusion. In both cases, antibody screening and identification by cell panels were negative. The incidence of delayed hemolytic transfusion reaction varies in the literature [19].

Two (2%) cases of non-specific transfusion reactions were reported: one occurred following platelet transfusion and another following whole blood transfusion. In both cases, the patients were apprehensive about blood transfusion.

Our retrospective analysis of transfusion reaction trends may serve as a reference when implementing preventive strategies to improve transfusion safety. Doctors, nurses, and paramedical staff must be adequately trained and sufficiently knowledgeable to report major and minor reactions, as this approach will lead to a better hemovigilance system.

Study limitations

The current study was dependent on the reporting of transfusion reactions, and there was under-reporting as many mild reactions, like allergic reactions, were not reported. The type of transfusion reactions department-wise could not be analyzed.

## Conclusions

The study concluded that there is a low incidence of transfusion reactions compared to various studies in literature, probably due to under-reporting by healthcare professionals. This may be due to a lack of awareness and knowledge about various presentations of transfusion reactions. Various factors leading to under-reporting should be analyzed and rectified. This emphasizes the need to conduct continuous medical and educational programs (CMEs) on the Hemovigilance System targeting new cadres (interns, faculties, post-graduates) to improve reporting practices. The study also provides insight into the fact that febrile transfusion reactions due to packed cell transfusion were the most common reactions reported, which can be prevented using leukocyte-reduced blood components. Leukocyte-reduced blood components can also prevent transmission of cytomegalovirus, Human leukocyte antigen (HLA) alloimmunization, and platelet refractoriness among transfusion recipients. Hospital Transfusion Committees should emphasize the implementation of newer technologies like leukocyte-reducing products. Thus, standardized, high-quality, and safe transfusion of blood and blood products can be promoted for patients in need.

## Figures and Tables

**Figure 4:**
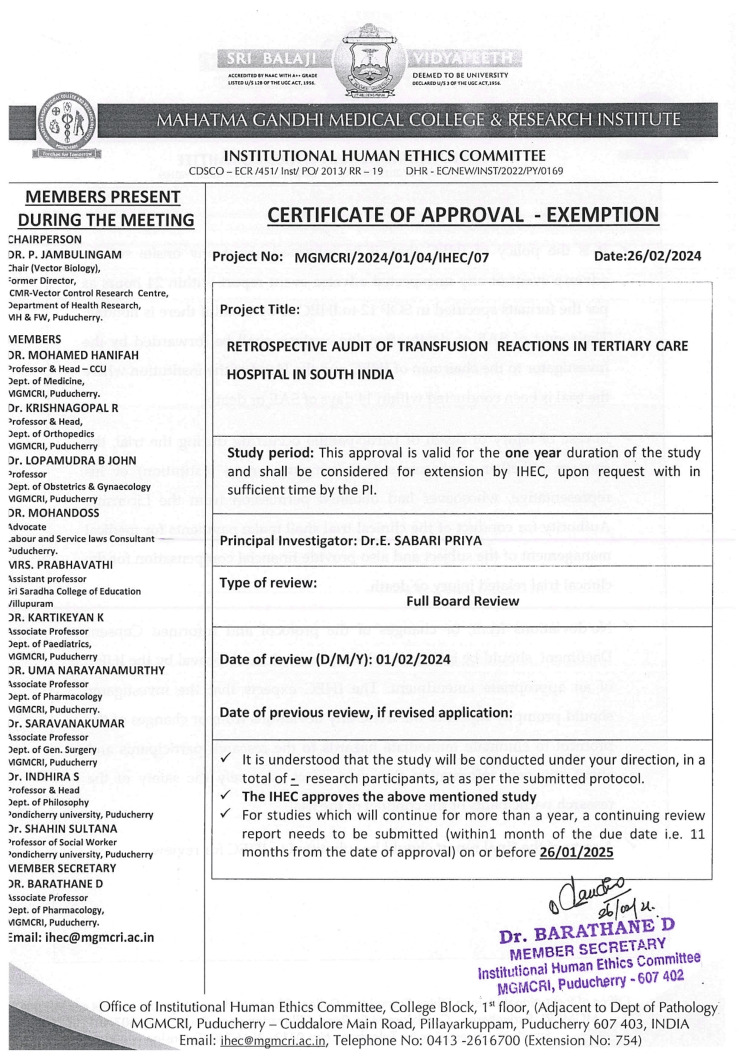
IHEC approval document
